# Lung sonography and recruitment in patients with early acute respiratory distress syndrome: A pilot study

**DOI:** 10.1186/cc10338

**Published:** 2011-08-04

**Authors:** Konstantinos Stefanidis, Stavros Dimopoulos, Elli-Sophia Tripodaki, Konstantinos Vitzilaios, Panagiotis Politis, Ploutarchos Piperopoulos, Serafim Nanas

**Affiliations:** 1 Department of Radiology, Evaggelismos Hospital, Ipsilantou 45-47, 10676, Athens, Greece; 2 1st Critical Care Medicine Department, Evaggelismos Hospital, NKUA, Ipsilantou 45-47, 10676, Athens, Greece

## Abstract

**Introduction:**

Bedside lung sonography is a useful imaging tool to assess lung aeration in critically ill patients. The purpose of this study was to evaluate the role of lung sonography in estimating the nonaerated area changes in the dependent lung regions during a positive end-expiratory pressure (PEEP) trial of patients with early acute respiratory distress syndrome (ARDS).

**Methods:**

Ten patients (mean ± standard deviation (SD): age 64 ± 7 years, Acute Physiology and Chronic Health Evaluation II (APACHE II) score 21 ± 4) with early ARDS on mechanical ventilation were included in the study. Transthoracic sonography was performed in all patients to depict the nonaerated area in the dependent lung regions at different PEEP settings of 5, 10 and 15 cm H_2_O. Lung sonographic assessment of the nonaerated lung area and arterial blood gas analysis were performed simultaneously at the end of each period. A control group of five early ARDS patients matched for APACHE II score was also included in the study.

**Results:**

The nonaerated areas in the dependent lung regions were significantly reduced during PEEP increases from 5 to 10 to 15 cm H_2_O (27 ± 31 cm^2 ^to 20 ± 24 cm^2 ^to 11 ± 12 cm^2^, respectively; *P *< 0.01). These changes were associated with a significant increase in arterial oxygen partial pressure (74 ± 15 mmHg to 90 ± 19 mmHg to 102 ± 26 mmHg; *P *< 0.001, respectively). No significant changes were observed in the nonaerated areas in the dependent lung regions in the control group.

**Conclusions:**

In this study, we show that transthoracic lung sonography can detect the nonaerated lung area changes during a PEEP trial of patients with early ARDS. Thus, transthoracic lung sonography might be considered as a useful clinical tool in the management of ARDS patients.

## Introduction

Acute respiratory distress syndrome (ARDS) is a clinical syndrome that often occurs in critically ill patients. It is a nonspecific response of the lung to injury due to a pulmonary or extrapulmonary insult. Specifically, it is characterized by the presence of diffuse lung inflammation, high permeability-type pulmonary oedema and massive loss of lung aeration in dependent lung regions and is associated with severe hypoxemia and a high mortality rate [1-3].

Patients with ARDS invariably require mechanical ventilation to decrease the work of breathing and to improve oxygen transport. An improvement in oxygenation can be obtained in many patients by an increase in positive end-expiratory pressure (PEEP), a strategy that was initially proposed in the first description of ARDS about 40 years ago [4]. PEEP is applied in patients with ARDS to avoid end-expiratory lung derecruitment and to improve oxygenation by increasing lung aeration.

Lung assessment is a frequent concern in critically ill patients with ARDS. After the first description of the syndrome [4], chest radiography was the only available technique for daily lung imaging. In chest radiography, the early stage of ARDS was usually recognized by the presence of bilateral alveolar infiltrates [5]. The invention of computed tomography (CT) has provided more precise information of the injured lung, illustrating the symmetric or asymmetric ground-glass opacification with the simultaneous presence of gravity-dependent atelectasis in ARDS patients [6-9]. Previous studies have described the role of the single juxtadiaphragmatic CT scan of the nonaerated lung parenchyma (Gattinoni's method) [10,11] and the CT assessment of the poorly aerated and nonaerated lung regions of the entire lung in ARDS patients [12]. In these studies, it was shown that a CT scan can detect the recruitment of previously nonaerated alveoli during PEEP increases.

CT is considered the reference test for assessing lung parenchyma in patients with ARDS, but it involves high irradiation and requires transportation of the critically ill patient to the department of radiology. These limitations make lung ultrasound (US) an attractive alternative to CT to assess lung morphology [13]. US is a noninvasive, radiation-free technique that is widely used in the ICU setting [14,15]. In patients on mechanical ventilation, US can be considered a reliable method to detect nonaerated lung regions [16]. Previous studies have shown the utility of US in the detection and quantification of lung recruitment via a transesophageal approach [17-19] and only recently via a transthoracic approach [20].

Our aim in this study was to evaluate transthoracic lung US as a clinical tool in the assessment of the nonaerated areas in the dependent right lung regions during a PEEP trial and to examine the relationship between the potentially recruitable lung as indicated by lung US and arterial oxygen partial pressure (PaO_2_).

## Materials and methods

### Population study

This prospective observational study of consecutive patients was conducted over an eight-month period from September 2009 to April 2010. The inclusion criteria of the study consisted of patients under mechanical ventilation admitted to the ICU with a stay ≥48 hours who met the standard criteria for ARDS. Specifically, the criteria were a ratio of PaO_2 _to the fraction of inspired oxygen (PaO_2_/FiO_2_) <200, the presence of bilateral pulmonary infiltrates on the chest radiograph and no clinical evidence of left atrial hypertension [1]. Patients' clinical condition was evaluated on the basis of the Acute Physiology and Chronic Health Evaluation II (APACHE II) score. The study's exclusion criteria were the presence of subcutaneous emphysema, severe obesity, intracranial hypertension, pregnancy and the absence of nonaerated lung regions assessed sonographically. We also excluded patients who could not maintain arterial oxygen saturation ≥85% during PEEP decreases. The included patients' baseline characteristics are shown in Table 1. All patients were sedated with propofol and/or midazolam and received vasopressor support as required. Tracheostomy was present in one patient. A control group of five early ARDS patients matched for APACHE II score was also included in the study. All enrolled patients were sonographically examined within 48 hours of the onset of ARDS. Informed consent to participate in this study was obtained from the patients' relatives as approved by the Scientific Council and the Ethics Committee of our institution.

**Table 1:** Baseline characteristics of all patients with ARDS enrolled in the study^a^

Characteristics	Data									
Patient	1	2	3	4	5	6	7	8	9	10
Age, years	62	59	62	57	75	58	57	62	66	78
APACHE II score	17	18	18	30	23	19	23	16	22	24
Disease	Postsurgical	Trauma	Postsurgical	Trauma	Trauma	Trauma	Sepsis/septic shock	Postsurgical	Haematological disease	Postsurgical
ARDS	Secondary	Primary	Secondary	Primary	Secondary	Secondary	Secondary	Secondary	Primary	Secondary
LISS	2.6	2.6	3	3.3	3.3	2.6	2.6	2.3	2.6	2.6
ICU day	7	5	3	3	6	7	3	13	4	11
PaO_2_/FiO_2_	145	106	189	61	96	119	132	198	142	162
FiO_2_	0.6	0.9	0.8	1	0.8	0.6	0.7	0.6	0.7	0.6
PaCO_2_	47	53	51	55	47	40	40	44	75	46
Heart rate, beats/minute	80	100	66	79	83	89	100	63	108	76
MAP, mmHg	68	94	80	65	74	70	70	79	72	68

^a^APACHE II, Acute Physiology and Chronic Health Evaluation II; LISS, Lung Injury Severity Score; PaO_2_, arterial oxygen partial pressure; FiO_2_, fraction of inspired oxygen; PaCO_2_, carbon dioxide partial pressure; MAP, mean arterial pressure.

### Design of the study

In all patients, PEEP settings of 5, 10 and 15 cm H_2_O were applied. The first assessment was performed at the baseline PEEP level that had been chosen as appropriate by the clinician. Consequently, reassessment was performed at the different PEEP levels (increased or reduced by 5 cm H_2_O). An expert ICU clinical investigator who participated in the study made the choice of the different PEEP settings. Each PEEP level was maintained for ≥20 minutes. All patients were under mechanical ventilation set at the volume assist-control mode. The tidal volume was set at 6 to 8 mL/kg, and the respiratory rate was adjusted to achieve a pH >7.25. FiO_2 _levels ranged from 0.6 to 1.0, depending on arterial gas analysis, to allow arterial oxygen saturation >90% A lung recruitment manoeuvre was performed in patients when their clinicians considered it necessary. No lung recruitment manoeuvre was performed during the duration of the study. The selected settings of ventilation and FiO_2_, with the exception of PEEP, remained unchanged during the study period. The nonaerated areas in the dependent right lung regions were calculated, and arterial blood gas analysis was simultaneously recorded at the end of each PEEP setting (5, 10 and 15 cm H_2_O). The control group of early ARDS patients underwent lung sonographic evaluation and arterial blood gas analysis before and one hour after initial evaluation without PEEP changes.

### Lung ultrasound

**Figure 1:**
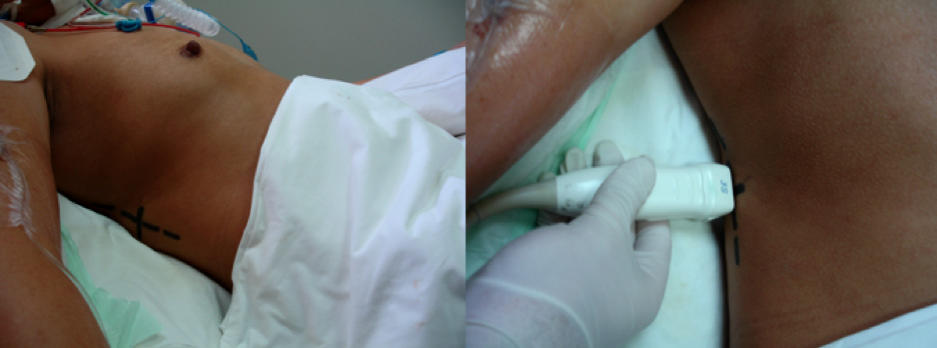
**Photograph of the probe's position in the posterior-axillary line perpendicular to the skin without angulation**. The position is marked on the skin to ensure reproducibility.

**Figure 2:**
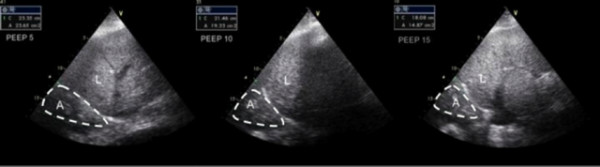
**An example of sonographic measurement of the nonaerated lung area at 5, 10 and 15 cm H_2_O positive end-expiratory pressure at the same posterior-axillary line in a patient with acute respiratory distress syndrome**. A = nonaerated lung area, L = liver.

Lung US was performed by one expert radiologist using a US system (Vivid 7; GE Healthcare, Wauwatosa, Wisconsin, U.S.A.) equipped with a sector array probe (1.5 to 3.8 MHz). The investigator who performed the lung US was blinded to the arterial gas analysis results and the PEEP values, as those were chosen by the ICU clinician. All patients were examined while in the semirecumbent position. All the measurements taken were of the nonaerated areas of the dependent regions of the right lung. First, the operator located the diaphragm. The probe was positioned longitudinally along the posterior-axillary line, perpendicular to the skin and without angulation to depict the nonaerated lung region (Figure 1). The position was marked for the next measurements at the same intercostal space. The nonaerated lung area and pleural effusion in the dependent and dorsal lung regions were located. They were sonographically defined as tissuelike and by an anechoic pattern, respectively. US was performed to depict the nonaerated areas in the dependent lung regions during PEEP settings of 5, 10, and 15 cm H_2_O (Figure 2). In all patients, the nonaerated area in the dependent lung region was observed at the same position of the probe during end expiration. US images were downloaded and saved on the hard disk of a personal computer. The results for all patients were analyzed at the end of the study. Each density area was outlined manually by two independent radiologists and was calculated using planimetry. The two independent radiologists were blinded to the arterial gas analysis results and PEEP values throughout the study.

### Arterial gas analysis

Arterial blood gas analysis was performed at all different PEEP changes to measure PaO_2 _and carbon dioxide partial pressure (ABL800 FLEX™; Radiometer Medical ApS, Copenhagen, Denmark). All measurements were performed simultaneously with the sonographic measurements of the nonaerated areas of the dependent lung regions.

### Statistical analysis

Continuous variables are presented as means ± standard deviations (SDs). Group means of continuous variables were compared by unpaired Student's *t*-test. Repeated-measures analysis of variance was used for the statistical evaluation of the within-group differences during the PEEP trial. A nonparametric Wilcoxon signed-rank test for continuous variables was performed, if required, for within-group comparisons after testing for normality curves by using the Kolmogorov-Smirnov test. Correlations between variables were obtained and tested by Pearson's correlation coefficient after being tested for normality curves. The lowest level for statistical significance was chosen as *P *< 0.05. Interobserver and intraobserver measurement variability, employing a limits of agreement method with Bland-Altman plots [21], was evaluated in all video images, including both patients who underwent the PEEP trial (*n *= 10) and the control group (*n *= 5). Systematic bias (the mean of the difference between the two measurements) and random error (the SD of the difference between the two measurements) were calculated.

## Results

A total of 15 patients with ARDS were evaluated for inclusion in our study. Of those, five patients were excluded from the study. The reasons for exclusion were nonaerated dependent lung regions not present during initial evaluation (*n *= 1), the presence of subcutaneous emphysema (*n *= 1) or severe obesity (*n *= 2). One other patient was excluded from the study because PEEP reduction to <10 cm H_2_O was not considered safe during evaluation (oxygen saturation <85%), and he could not complete the assessment.

**Figure 3:**
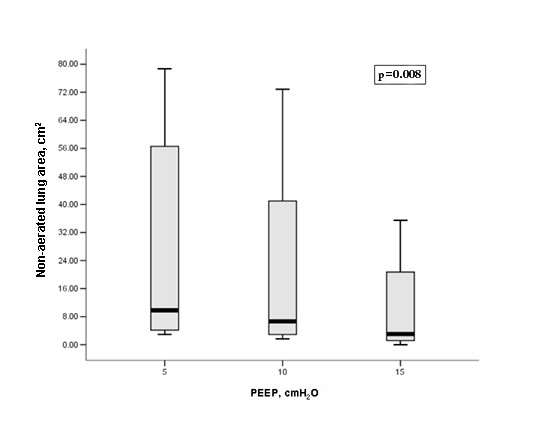
**Boxplot of the nonaerated lung areas at different positive end-expiratory pressure (PEEP) levels (5, 10 and 15 cm H_2_O) in ARDS patients**. {the upper and the lower boundary of the "box" (grey-shaded area) represents the 75^th ^and 25^th ^percentile of the data, the central black line of the " box" is the median value and the vertical lines indicate the maximum and the minimum values}

**Figure 4:**
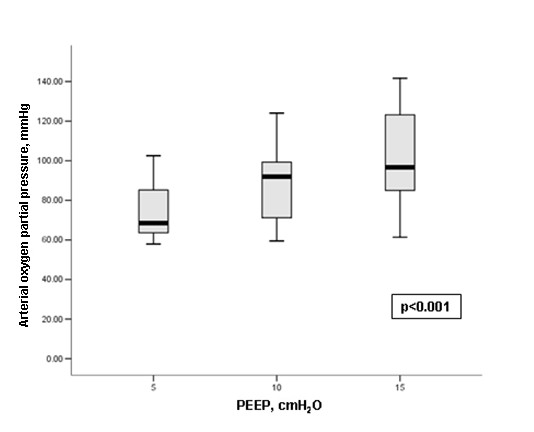
**Boxplot of the arterial oxygen partial pressure at different PEEP levels (5, 10 and 15 cm H_2_O) in ARDS patients**. {the upper and the lower boundary of the "box" (grey-shaded area) represents the 75^th ^and 25^th ^percentile of the data, the central black line of the " box" is the median value and the vertical lines indicate the maximum and the minimum values}

Ten patients with ARDS were ultimately enrolled in the study (Table 1). All patients presented a significant increase in PaO_2 _levels and PaO_2_/FiO_2 _ratios and parallel decreases in the nonaerated lung area as the PEEP level was increased from 5 to 15 cm H_2_O (Figures 3 and 4 and Table 2). An example of a nonaerated lung area reduction during PEEP increase is illustrated in Figure 1.

**Table 2:** Monitoring during PEEP changes in ARDS patients included in the study^a^

Measurement	PEEP 5 cm H_2_O (*N *= 10)	PEEP 10 cm H_2_O (*N *= 10)	PEEP 15 cm H_2_O (*N *= 10)	*P *value
Arterial blood gas measurements				
PaO_2_, mmHg	74 ± 15^b^	90 ± 19^c^	102 ± 26^d^	<0.001
PaCO_2_, mmHg	51 ± 11	50 ± 10	52 ± 12	NS (0.08)
pH	7.31 ± 0.09	7.32 ± 0.09^c^	7.3 ± 0.09^d^	0.002
SaO_2_, %	94 ± 3^e^	97 ± 3	97 ± 3^f^	<0.001
PaO_2_/FiO_2_	106 ± 35^e^	133 ± 43^c^	153 ± 57^f^	<0.001
Haemodynamic and ventilatory parameters				
SAP, mmHg	126 ± 18	120 ± 15	122 ± 19	NS (0.39)
DAP, mmHg	51 ± 11	50 ± 7	53 ± 10	NS (0.4)
MAP, mmHg	76 ± 10	74 ± 7	75 ± 11	NS (0.6)
HR, beats/minute	85 ± 14	83 ± 14	85 ± 14	NS (0.6)
PIP, cmH_2_O	34 ± 6	37 ± 10^c^	43 ± 12	NS (0.06)
MIP, cmH_2_O	12 ± 2^e^	18 ± 2^c^	22 ± 3^g^	<0.001
Lung sonographic evaluation				
Nonaerated lung area, cm^2^	27 ± 31^e^	20 ± 24^c^	11 ± 12^f^	0.008

^a^All data are means ± SD. PEEP, positive end-expiratory pressure; PaO_2_, arterial oxygen partial pressure; FiO_2_, fraction of inspired oxygen; PaCO_2_, carbon dioxide partial pressure; SaO_2_, oxygen saturation; SAP, systolic arterial pressure; DAP, diastolic arterial pressure; MAP, mean arterial pressure; HR, heart rate; PIP, peak inspiratory pressure; MIP, mean inspiratory pressure. ^b^*P *< 0.01 comparing PEEP 5 and 10 cm H_2_O for PaO_2 _measurements. ^c^*P *< 0.05 comparing PEEP 10 and 15 cm H_2_O for PaO_2_, pH, PaO_2_/FiO_2_, PIP, MIP and nonaerated lung area measurements. ^d^*P *< 0.01 comparing PEEP 5 and 15 cm H_2_O for PaO_2 _and pH measurements. ^e^*P *< 0.05 comparing PEEP 5 and 10 cm H_2_O for SaO_2_, PaO_2_/FiO_2_, MIP and nonaerated lung area measurements. ^f^*P *< 0.05 comparing PEEP 5 and 15 cm H_2_O for SaO_2_, PaO_2_/FiO_2 _and nonaerated lung area measurements. ^g^*P *< 0.001 comparing PEEP 5 and 15 cm H_2_O for MIP measurements.

No haemodynamic deterioration was noted during PEEP increase in terms of invasively measured mean systolic and diastolic arterial pressure and mean heart rate. There were increases in peak inspiratory pressure and mean inspiratory pressure at the PEEP level of 15 cm H_2_O compared to the PEEP level of 5 cm H_2_O (Table 2). Patients with nonaerated lung area reduction >60% during PEEP increase from 5 to 15 cm H_2_O had a higher initial APACHE II score (25 ± 4 versus 18 ± 2; *P *< 0.05). The degree of PaO_2 _increase did not significantly correlate with the degree of nonaerated lung area reduction (*r *= -0.2, *P *= 0.9). No changes in treatment were noted during the PEEP trial in all patients.

**Figure 5:**
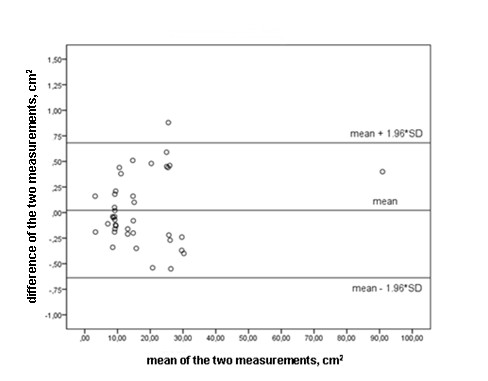
**Bland-Altman plot with 95% limits of agreement for intraobserver measurement variability (measurement of the non aerated dependent lung sonographic area, cm^2^)**.

**Figure 6:**
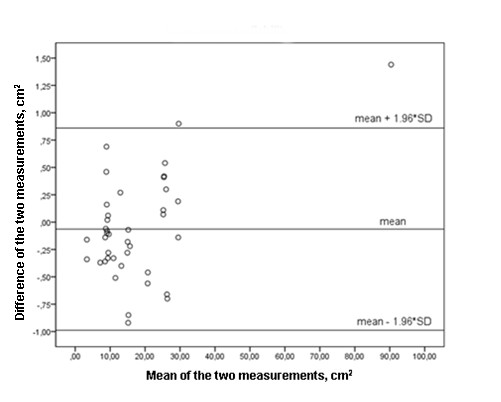
**Bland-Altman plot with 95% limits of agreement for interobserver measurement variability (measurement of the non aerated dependent lung sonographic area, cm^2^)**.

In the control group, no significant difference was found at baseline or one hour after lung assessment in PaO_2 _measurements (72 ± 15 mmHg to 72 ± 15 mmHg) and the nonaerated lung areas in the dependent regions (15 ± 19 cm^2 ^to 15 ± 19 cm^2^). The mean PEEP level of the control group was 13.4 ± 4.2 cm H_2_O. The control group matched the patient group on APACHE II score severity (23 ± 6 versus 21 ± 4; *P *= 0.6). US image analysis showed systematic bias and random error 0.02 ± 0.34 cm^2 ^for intraobserver measurement variability and -0.06 ± 0.47 cm^2 ^for interobserver measurement variability (Figures 5 and 6).

## Discussion

In this study, lung US was performed to observe real-time monitoring of the nonaerated area changes in the dependent lung regions during a PEEP trial. The results clearly show that the nonaerated lung area was significantly reduced during PEEP increases from 5 to 15 cm H_2_O and that these changes were accompanied by a significant increase in PaO_2_. To our knowledge, this study is the first to show in real time the sonographic changes of the nonaerated areas of the dependent right lung regions using a transthoracic approach during a PEEP trial.

In a previous study, Tsubo *et al*. [17,18] demonstrated that it is possible to estimate the density area of the dependent left lung regions in patients with acute lung injury (ALI) or ARDS using transesophageal echocardiography. In their study, it was also possible to observe changes in lung density areas during application of PEEP. The same research group subsequently demonstrated that transesophageal echocardiography can detect density area changes in ARDS patients in the prone position [19]. In a recent interesting case study by Gardelli *et al*. [22], the use of transthoracic US in identifying recruitable lung density areas was reported. These authors managed to show the recruitment of consolidated areas with lower PEEP in a female patient with ARDS in the prone position. Other studies have demonstrated the diagnostic accuracy of lung US in detecting alveolar consolidation in critically ill patients in comparison with the CT, which is considered the "gold standard" test, [23]. In an important recently published study, Bouhemad *et al*. [20] investigated the role of bedside US assessment of PEEP-induced lung recruitment. In their study, 40 patients with ARDS and/or ALI were prospectively enrolled and the pressure-volume curve method was assessed. The results of that study showed that PEEP-induced lung recruitment can be adequately estimated with bedside US (as assessed by US reaeration score). Other studies have reported the clinical utility of lung US in assessing lung aeration in cardiogenic and high-altitude pulmonary oedema after medical treatment of patients with acute decompensated heart failure, patients undergoing haemodialysis and patients with community-acquired and ventilator-associated pneumonia [24-29]. The findings of our study extend the role of bedside lung US in ARDS patients. More specifically, we provide confirming evidence that lung US can detect the nonaerated lung area reduction in all ARDS patients during PEEP increases.

CT is considered a valid technique to estimate lung recruitment in ARDS patients by quantifying the amount of tissue according to the different lung recruitment manoeuvres (the so-called "potentially recruitable lung") [12]. Gattinoni *et al*. [30] demonstrated that the percentage of "potentially recruitable lung" estimated by CT in ARDS is extremely variable and strongly associated with the response to PEEP. This technique can provide dynamic whole-lung scanning of anatomical and functional lung morphology and, through lung recruitment manoeuvres, can be used to optimize alveolar recruitment in patients with ALI and/or ARDS [31]. Although CT has an important role in lung recruitment assessment and can be used to detect several complications during mechanical ventilation, it has some significant limitations. The need to transport the critically ill patient to the department of radiology, the high irradiation exposure, the unavailability of CT and the high cost are some limiting factors that make the research of other alternative methods for lung recruitment assessment in ARDS patients necessary.

US is considered a technique with a wide range of applications in the ICU setting [32]. Its safety and portability allow for its use at the bedside to provide rapid and detailed information regarding pathology of the thorax and abdomen. Despite initial technical limitations, lung US is now used in an increasing number of pathological situations, such as pneumonia, atelectasis, interstitial-alveolar syndrome, pulmonary embolism, pneumothorax and pleural effusion [23,33-38].

Lung US allows evaluation of lung aeration in patients with ARDS and ALI at the bedside [20,39,40] and can detect atelectasis and/or consolidation in patients under mechanical ventilation [16,17]. The presence of a nonaerated lung area adjacent to the visceral pleura is necessary to visualize a potentially recruitable lung region by lung US, limiting assessment in patients with ARDS mainly due to extrapulmonary causes [7].

However, lung sonography may allow continuous monitoring of the nonaerated dependent lung regions, avoiding derecruitment in clinical practice. A potential future clinical implication of lung US might be the prevention of high PEEP levels in patients with a small amount of potentially recruitable lung areas ("nonresponders"), minimizing ventilator-associated lung injury. For this purpose, further studies are required to determine the role of US as an imaging tool during PEEP or other recruitment manoeuvres and to compare it to other standard techniques such as CT.

There are some potential limitations of the present study. This study consisted of a small sample size that did not allow us to stratify ARDS patients according to the rate of the nonaerated lung area reduction after PEEP increase ("responders" versus "nonresponders"). Limitations are also associated with the methodology of lung US. Longitudinal scanning at the level of the diaphragm does not assess, in some cases, the real extension of the nonaerated lung region. In addition, in such cases, the increase in PEEP level may be correlated to the movement of the dependent lung regions along a cephalocaudal axis. In these cases, the reduction of the nonaerated lung area can be partially overestimated and may not be correlated exclusively with lung recruitment. US scanning is also limited to the detection of the recruitable lung of the right dependent lung regions and not of the whole lung. This limits the detection of possible lung overinflation and may underestimate PEEP-induced lung recruitment in the anterior and lateral parts of the lung. The recently published study by Bouhemad *et al*. [20] described the role of lung US in the assessment of PEEP-induced recruitment in all ARDS patients by using four patterns of aeration score (including B-lines assessment). However, it seems that the simplicity of lung US evaluation performed in the present study confers a significant and complementary role during lung recruitment compared to that described by Bouhemad *et al*. It can be considered as a simple qualitative method by which the clinician can recognize PEEP-induced lung recruitment in the nonaerated dependent lung areas. Though US is an operator-dependent methodology compared to the CT scan, we managed to provide low measurement variability of the method to detect lung nonaerated regions. Sonographic imaging of lung dependent regions was not possible in patients with subcutaneous emphysema and severe obesity, and not all initially evaluated patients presented with lung atelectatic areas. In our study, we restricted lung sonographic assessment at PEEP settings of 5, 10 and 15 cm H_2_O; however, these PEEP changes were sufficient to demonstrate significant differences in nonaerated lung areas in all patients.

## Conclusions

In this study, we have shown that transthoracic lung US can detect nonaerated lung area reduction during PEEP increases from 5 to 15 cm H_2_O in patients with ARDS. Further studies are needed to determine whether transthoracic US assessment of dependant nonaerated lung areas is accurate to quantify PEEP-induced lung recruitment.

## Key messages

• Lung assessment is a frequent concern in critically ill patients with ARDS.

• Lung US detected nonaerated lung area changes in the dependent lung regions during a PEEP trial of patients with early ARDS.

• Lung US showed that the nonaerated areas of the dependent lung regions were reduced during PEEP increases from 5 to 10 to 15 cm H_2_O.

• Lung US was found to be a promising, simple bedside tool in the evaluation of lung aeration in patients with ARDS during a PEEP trial.

## Abbreviations

APACHE II: Acute Physiology and Chronic Health Evaluation II; ARDS: acute respiratory distress syndrome; CT: computed tomography; FiO_2_: fraction of inspired oxygen; MIP: mean inspiratory pressure; PaO_2_: arterial oxygen partial pressure; PEEP: positive end-expiratory pressure; PIP: peak inspiratory pressure; US: ultrasound.

## Competing interests

The authors declare that they have no competing interests.

## Authors' contributions

All authors contributed substantially to the submitted work and read and approved the final manuscript. In particular, KS conceptualized this study and was responsible for the design, analysis, data interpretation and drafting of the manuscript. SD participated in the design of the study, analysed the data and revised the manuscript. EST and KV participated in data collection and interpretation. PPo and PPi participated in the design and revised the manuscript. SN critically revised the manuscript and provided final approval for its publication.

## References

[B1] Bernard GR Artigas A Brigham KL Carlet J Falke K Hudson L Lamy M Legall JR Morris A Spragg R The American-European Consensus Conference on ARDS: definitions, mechanisms, relevant outcomes, and clinical coordination Am J Respir Crit Care Med 1994 149 818 824 7509706 10.1164/ajrccm.149.3.7509706

[B2] Roupie E Lepage E Wysocki M Fagon JY Chastre J Dreyfuss D Mentec H Carlet J Brun-Buisson C Lemaire F Brochard L Prevalence, etiologies and outcome of the acute respiratory distress syndrome among hypoxemic ventilated patients. SRLF Collaborative Group on Mechanical Ventilation. Société de Réanimation de Langue Française Intensive Care Med 1999 25 920 929 10.1007/s001340050983 10501746

[B3] Luhr O Antonsen K Karlsson M Aardal S Thosteinsson A Frostell CG Bonde J Incidence and Mortality after acute respiratory failure and acute respiratory distress syndrome in Sweden, Denmark, and Iceland. The ARF Study Group Am J Respir Crit Care Med 2000 162 332 333 10351930 10.1164/ajrccm.159.6.9808136

[B4] Ashbaugh DG Bigelow DB Petty TL Levine BE Acute respiratory distress in adults Lancet 1967 2 319 3234143721

[B5] Winer-Muram HT Rubin SA Ellis JV Jennings SG Arheart KL Wunderink RG Leeper KV Meduri GU Pneumonia and ARDS in patients receiving mechanical ventilation: diagnostic accuracy of chest radiography Radiology 1993 188 479 485 8327701 10.1148/radiology.188.2.8327701

[B6] Tagliabue M Casella MC Zincone GE Fumagalli R Salvini E CT and chest radiography in the evaluation of adult respiratory distress syndrome Acta Radiologica 1994 35 230 234 8192958

[B7] Goodman L Fumagalli R Tagliabue P Tagliabue M Ferrario M Gattinoni L Pesenti A Adult respiratory distress syndrome due to pulmonary and extrapulmonary causes: CT, clinical, and functional correlations Radiology 1999 213 545 552 10551239 10.1148/radiology.213.2.r99nv42545

[B8] Rouby JJ Puybasset L Nieszkowska A Lu Q Acute respiratory distress syndrome: lessons from computed tomography of the whole lung Crit Care Med 2003 31 S285 S295 10.1097/01.CCM.0000057905.74813.BC 12682454

[B9] Desai S Wells A Suntharalingam G Rubens M Evans T Hansell D Acute respiratory distress syndrome caused by pulmonary and extrapulmonary injury: a comparative CT study Radiology 2001 218 689 693 11230641 10.1148/radiology.218.3.r01mr31689

[B10] Gattinoni L D'Andrea L Pelosi P Vitale G Pesenti A Fumagalli R Regional effects and mechanism of positive end-expiratory pressure in early adult respiratory distress syndrome JAMA 1993 269 2122 2127 A published erratum appears in *JAMA *1993, **270:**1814 10.1001/jama.269.16.2122 8468768

[B11] Gattinoni L Pelosi P Crotti S Valenze F Effects of positive end-expiratory pressure on regional distribution of tidal volume and recruitment in adult respiratory distress syndrome Am J Respir Crit Care Med 1995 151 1807 1814 7767524 10.1164/ajrccm.151.6.7767524

[B12] Malbouisson LM Muller JC Constantin JM Lu Q Puybasset L Rouby JJ Computed tomography assessment of positive end-expiratory pressure-induced alveolar recruitment in patients with acute respiratory distress syndrome Am J Respir Crit Care Med 2001 163 1444 1450 11371416 10.1164/ajrccm.163.6.2005001

[B13] Lichtenstein D Goldstein I Mourgeon E Cluzel P Grenier P Rouby JJ Comparative diagnostic performances of auscultation, chest radiography, and ultrasonography in acute respiratory distress syndrome Anesthesiology 2004 100 9 15 10.1097/00000542-200401000-00006 14695718

[B14] Bouhemad B Zhang M Lu Q Rouby JJ Clinical review: bedside lung ultrasound in critical care practice Crit Care 2007 11 205 10.1186/cc5668 17316468 PMC2151891

[B15] Stefanidis K Dimopoulos S Nanas S Basic principles and current applications of lung ultrasonography in the intensive care unit Respirology 2011 16 249 256 10.1111/j.1440-1843.2010.01885.x 20969673

[B16] Yang JX Zhang M Liu ZH Ba L Gan JX Xu SW Detection of lung atelectasis/consolidation by ultrasound in multiple trauma patients with mechanical ventilation Clin Ultrasound J 2009 1 13 16 10.1007/s13089-009-0003-x

[B17] Tsubo T Sakai I Suzuki A Okawa H Ishihara H Matsuki A Density detection in dependent left lung region using transesophageal echocardiography Anesthesiology 2001 94 793 798 10.1097/00000542-200105000-00017 11388530

[B18] Tsubo T Yatsu Y Suzuki A Iwakawa T Okawa H Ishihara H Matsuki A Daily changes of the area of density in the dependent lung region: evaluation using transesophageal echocardiography Intensive Care Med 2001 27 1881 1886 10.1007/s00134-001-1115-3 11797023

[B19] Tsubo T Yatsu Y Tanabe T Okawa H Ishihara H Matsuki A Evaluation of density area in dorsal lung region during prone position using transesophageal echocardiography Crit Care Med 2004 32 83 87 10.1097/01.CCM.0000104944.18636.B2 14707563

[B20] Bouhemad B Brisson H Le-Guen M Arbelot C Lu Q Rouby JJ Bedside ultrasound assessment of positive end-expiratory pressure-induced lung recruitment Am J Respir Crit Care Med 2011 183 341 347 10.1164/rccm.201003-0369OC 20851923

[B21] Bland JM Altman DG Statistical methods for assessing agreement between two methods of clinical measurements Lancet 1986 1 307 310 2868172

[B22] Gardelli G Feletti F Gamberini E Bonarelli S Nanni A Mughetti M Using sonography to assess lung recruitment in patients with acute respiratory distress syndrome Emerg Radiol 2009 16 219 221 10.1007/s10140-008-0734-1 18830644

[B23] Lichtenstein DA Lascols N Mezière G Gepner A Ultrasound diagnosis of alveolar consolidation in the critically ill Intensive Care Med 2004 30 276 281 10.1007/s00134-003-2075-6 14722643

[B24] Agricola E Bove T Oppizzi M Marino G Zangrillo A Margonato A Picano E "Ultrasound comet-tail images": a marker of pulmonary edema: a comparative study with wedge pressure and extravascular lung water Chest 2005 127 1690 1695 10.1378/chest.127.5.1690 15888847

[B25] Fagenholz PJ Gutman JA Murray AF Noble VE Thomas SH Harris NS Chest ultrasonography for the diagnosis and monitoring of high-altitude pulmonary edema Chest 2007 131 1013 1018 10.1378/chest.06-1864 17426204

[B26] Volpicelli G Caramello V Cardinale L Mussa A Bar F Francisco MF Bedside ultrasound of the lung for the monitoring of acute decompensated heart failure Am J Emerg Med 2008 26 585 591 10.1016/j.ajem.2007.09.014 18534289

[B27] Noble VE Murray AF Capp R Sylvia-Reardon MH Steele DJR Liteplo A Ultrasound assessment for extravascular lung water in patients undergoing hemodialysis: time course for resolution Chest 2009 135 1433 1439 10.1378/chest.08-1811 19188552

[B28] Bouhemad B Liu ZH Arbelot C Zhang M Ferarri F Le-Guen M Girard M Lu Q Rouby JJ Ultrasound assessment of antibiotic-induced pulmonary reaeration in ventilator-associated pneumonia Crit Care Med 2010 38 84 92 10.1097/CCM.0b013e3181b08cdb 19633538

[B29] Reissig A Kroegel C Sonographic diagnosis and follow-up of pneumonia: a prospective study Respiration 2007 74 537 547 10.1159/000100427 17337882

[B30] Gattinoni L Caironi P Cressoni M Chiumello D Ranieri VM Quintel M Russo S Patroniti N Cornejo R Bugedo G Lung recruitment in patients with acute respiratory distress syndrome N Engl J Med 2006 354 1775 1786 10.1056/NEJMoa052052 16641394

[B31] Bugedo G Bruhn A Hernández G Rojas G Varela C Tapia JC Castillo L Lung computed tomography during a lung recruitment maneuver in patients with acute lung injury Intensive Care Med 2003 29 218 225 12536272 10.1007/s00134-002-1618-6

[B32] Lichtenstein D Axler O Intensive use of general ultrasound in the intensive care unit: prospective study of 150 patients Intensive Care Med 1993 9 353 355 8227728 10.1007/BF01694712

[B33] Lichtenstein DA Lascols N Prin S Mezière G The "lung pulse": an early ultrasound sign of complete atelectasis Intensive Care Med 2003 29 2187 2192 10.1007/s00134-003-1930-9 14557855

[B34] Lichtenstein D Mezière G Biderman P Gepner A Barre O The comet-tail artifact: an ultrasound sign of alveolar-interstitial syndrome Am J Respir Crit Care 1997 156 1640 1646 10.1164/ajrccm.156.5.96-07096 9372688

[B35] Mathis G Blank W Reißig A Lechleitner P Reuß J Schuler A Beckl S Thoracic ultrasound for diagnosing pulmonary embolism: a prospective multicenter study of 352 patients Chest 2005 128 1531 1538 10.1378/chest.128.3.1531 16162754

[B36] Lichtenstein DA Mezière G Lascols N Biderman P Courret JP Gepner A Goldstein I Tenoudji-Cohen M Ultrasound diagnosis of occult pneumothorax Crit Care Med 2005 33 1231 1238 10.1097/01.CCM.0000164542.86954.B4 15942336

[B37] Balik M Plasil P Waldauf P Pazout J Fric M Otahal M Pachl J Ultrasound estimation of volume of pleural fluid in mechanically ventilated patients Intensive Care Med 2006 32 318 321 10.1007/s00134-005-0024-2 16432674

[B38] Roch A Bojan M Michelet P Romain F Bregeon F Papazian L Auffray JP Usefulness of ultrasonography in predicting pleural effusions >500 mL in patients receiving mechanical ventilation Chest 2005 127 224 232 10.1378/chest.127.1.224 15653988

[B39] Via G Lichtenstein D Mojoli F Rodi G Neri L Storti E Klersy C Iotti G Braschi A Whole lung lavage: a unique model for ultrasound assessment of lung aeration changes Intensive Care Med 2010 36 999 1007 10.1007/s00134-010-1834-4 20221746

[B40] Arbelot C Ferrari F Bouhemad B Rouby JJ Lung ultrasound in acute respiratory distress syndrome and acute lung injury Curr Opin Crit Care 2008 14 70 74 10.1097/MCC.0b013e3282f43d05 18195629

